# Transformative equality: Court accommodations for South African citizens with severe communication disabilities

**DOI:** 10.4102/ajod.v9i0.651

**Published:** 2020-04-01

**Authors:** Robyn M. White, Juan Bornman, Ensa Johnson, Karen Tewson, Joan van Niekerk

**Affiliations:** 1 Centre for Augmentative and Alternative Communication, University of Pretoria, Pretoria, South Africa; 2 National Prosecuting Authority, Pretoria, South Africa; 3 Private, Durban, South Africa

**Keywords:** communication disability, access to justice, human rights, South Africa, court accommodations

## Abstract

**Background:**

Persons with disabilities are generally at greater risk of experiencing violence than their peers without a disability. Within the sphere of disability, individuals with severe communication disabilities are particularly vulnerable and have an increased risk of being a victim of abuse or violence and typically turn to their country’s criminal justice system to seek justice. Unfortunately, victims with disabilities are often denied fair and equal treatment before the court. Transformative equality should be pursued when identifying accommodations in court for persons with communication disabilities, as the aim should be to enable such individuals to participate equally in court, without barriers and discrimination.

**Objectives:**

This research aimed to identify court accommodations recommended by legal experts, which could assist individuals with severe communication disabilities in the South African court.

**Method:**

A qualitative design was used to conduct a discussion with a panel of legal experts.

**Results:**

Using Article 13 (Access to Justice) of the Convention on the Rights of Persons with Disabilities (CRPD) as a human rights framework, four themes were identified: equality, accommodations, participation and training of professionals.

**Conclusion:**

Foreign and national law clearly prohibits discrimination against persons with communication disabilities because of their disability and state that they should be given fair and equal access to the court system. For transformative equality to be achieved, certain rules and laws need to be changed to include specific accommodations for persons with communication disabilities so that they may be enabled to participate effectively in court in the criminal justice system.

## Introduction and background

Persons with disabilities are at greater risk of experiencing violence than their peers without a disability.

Globally, children with disabilities are three to four times more likely to experience violence than their peers without disability (World Health Organization 2015). Recently, a South African study also estimated that children with disabilities were 1.5 and 2.1 times more at risk of sexual abuse than their peers without a disability (Artz et al. 2016). In an American study that compared 9086 women with and without a disability, results showed that 39% of the women who had been raped in the 12 months preceding the survey had a disability at the time of the rape (Basile, Breiding & Smith 2016). Another American study that reported on 21 615 respondents and their victimisation found that 26.6% of women with disabilities reported sexual violence compared with 12.4% of women without disabilities (Mitra, Mouradian & Diamond 2011). This trend was also observed in American men, as 13.9% of men with disabilities reported sexual violence compared with 3.7% of men without disabilities (Mitra et al. 2011).

Within the sphere of disability, individuals with severe communication disabilities are particularly vulnerable and have an increased risk of becoming victims of abuse (Bornman, Bryen, Kershaw & Ledwabe 2011). This may be because of the fact that they are unable to shout or call for help, or because perpetrators often seek out vulnerable individuals who they perceive as being unable to verbalise their victimisation to family members or key legal role players such as the police and court officials (White, Bornman & Johnson 2015). For example, in a systematic review of 21 557 adults with disabilities, the prevalence of recent violence was 24.3% in persons with mental illnesses, 6.1% in those with intellectual impairments and 3.2% in those with non-specific impairments (Hughes et al. 2012). In another meta-analysis, from a total of 14 721 children with disabilities, the prevalence of recent violence was 26.7% for combined violence, 20.4% for physical violence and 13.7% for sexual violence (Jones et al. 2012).

Typically, persons without disabilities who were victims of violence or crime turn to their country’s criminal justice system to seek justice by reporting the crime to the police and testifying in a court against the accused perpetrator(s). This same process should be available to persons with disabilities (White & Msipa 2018).

However, persons with disabilities are often denied fair and equal treatment before the courts (Flynn 2013).

When persons with communication disabilities try to report their victimisation, the police – through ignorance of the disability – may often mistakenly decide that the victim will not meet the legal requirements of being a competent witness in court, and hence, they fail to proceed appropriately and lawfully (Archer & Hurley 2013; Viljoen 2018).

Equally important, offenders with intellectual and mental disabilities may also struggle with communication challenges, which could have a negative impact on their pursuit of access to justice (Capri et al. 2018).

Offenders with communication disabilities are also vulnerable to exploitation and being influenced and professionals in the court system should be aware of the vulnerabilities of this population (Capri et al. 2018).

Legal representatives of both victims and perpetrators must be able to respond appropriately to maintain the fairness and dignity of the court system (Salekin, Olley & Hedge 2010). Nonetheless, a comprehensive focus on perpetrators is beyond the scope of this study.

The Convention on the Elimination of All Forms of Discrimination Against Women (CEDAW) jurisprudence is of use to understand equality and non-discrimination obligations in conditions of systemic power inequality (e.g. the court system) (United Nations 1988). The CEDAW Committee identified three types of obligations: formal equality (equal treatment as a matter of law), substantive equality (measures to equalise the enjoyment of human rights) and transformative equality (measures to remove the causes of inequality) (Minkowitz 2017). Formal equality is needed to have equal status as members of society, substantive equality is needed to proactively redistribute power and resources, and transformative equality is needed to transform opportunities, institutions and systems so that they are no longer grounded in historically determined paradigms of power (Minkowitz 2017). For the purpose of this article, the focus will be on transformative equality.

Transformative equality recognises the need to change rules and laws to include different perspectives and not only dominant views and experiences (Goldschmidt 2017). As such, it targets certain structures and systems (including the court system) for change through introducing a variety of positive measures for persons with disabilities (Degener 2016). An international rights treaty that emphasises transformative equality for persons with disabilities is the Convention on the Rights of Persons with Disabilities (CRPD) (United Nations 2006).

The CRPD was inspired by international treaties to promote and support the human and legal rights of all persons with disabilities (United Nations 2006). To date, this treaty has been signed and ratified by 46 African counties including South Africa, who ratified it in 2007. Goldschmidt (2017) highlights the five principles of the CRPD which are equality, accessibility, autonomy, participation and inclusion. Furthermore, these principles of the CRPD reflect the four dimensions of substantive equality which are redressing disadvantage (the redistributive dimension); addressing stigma, stereotyping, prejudice and violence (the recognition dimension); facilitating voice and participation (the participative dimension); and accommodating difference, including through structural change (the transformative dimension) (Fredman 2005).

Article 13 of the CRPD specifically reports on ‘Access to Justice’ and states that:

[*A*]ll States Parties shall ensure effective access to justice for persons with disabilities on an equal basis with others, including through the provision of procedural and age-appropriate accommodations, in order to facilitate their effective role as direct and indirect participants, including as witnesses, in all legal proceedings. (United Nations 2006:11)

The provision of procedural and age-appropriate accommodations is distinguishable from the term ‘reasonable accommodation’ in that procedural accommodations are not limited by disproportionality (Committee on the Rights of Persons with Disabilities 2018). ‘Reasonable accommodations’ can be defined as appropriate modifications and adjustments not imposing a disproportionate or undue burden, where needed in a particular case, to ensure to persons with disabilities the enjoyment on an equal basis with others, of all human rights (United Nations 2006). Procedural accommodation is the recognition of different communication methods of persons with communication disabilities to be able to participate in court. Age-appropriate accommodations may consist of providing information about available mechanisms to bring complaints forward and using age-appropriate and simple language (Committee on the Rights of Persons with Disabilities 2018).

Article 13 further states that:

[*I*]n order to help to ensure effective access to justice for persons with disabilities, States Parties shall promote appropriate training for those working in the field of administration of justice. (United Nations 2006:11)

In addition, the Protocol to the African Charter on Human and Peoples’ Rights on the Rights of Persons with Disabilities in Africa was adopted in 2018 with South Africa being one of the signatories. In this protocol, Article 13 addresses the ‘Right to Access Justice’ and also highlights that state parties should ensure that persons with disabilities have access to justice on an equal basis with others, including through the provision of appropriate (age and gender) and procedural accommodations (African Union 2018).

In principle, South Africa has passed the relevant legislation that specifically accommodates victims with disabilities who need to access the court system and that allows equal participation in all legal proceedings. For example, Section 9 of the South African Constitution foregrounds equality and states that ‘[e]veryone is equal before the law and has the right to equal protection and benefit of the law, including persons with disabilities’. *The Promotion of Equality and Prevention of Unfair Discrimination Act 4 of 2000* likewise emphasises that no one should be discriminated against on the ground of disability and underscores that ‘failing to eliminate obstacles that unfairly limit or restrict persons with disabilities from enjoying equal opportunities or failing to take steps to reasonably accommodate the needs of such persons’ is unconstitutional. Persons with a communication disability may therefore not be discriminated against in a court of law because of their inability to communicate, and key role players in the court system should provide court accommodations to assist such individuals to be able to communicate and testify in court (The Constitution of the Republic of South Africa, 1996).

Despite existing foreign and national legislation, persons with communication disabilities and their families still find it difficult and overwhelming to access and participate effectively in the criminal justice system, irrespective of being a witness or an alleged perpetrator (Bornman et al. 2016). This could be because of the limited and constrained resources, accommodations and support offered to persons with communication disabilities who need to access the court system (Fitzsimons 2016). Flynn (2016) highlights three distinct inaccessible features in the court system that unfairly affect persons with disabilities: (1) the physical infrastructure that refers to architectural features such as staircases instead of ramps that act as environmental barriers; (2) procedural barriers that refer to when persons with disabilities cannot understand the court procedures and communicate effectively with the key role players in the court system; and (3) evidentiary barriers that refer to non-adapted rules of evidence and procedures to facilitate effective participation of persons with communication disabilities as witnesses.

In an attempt to overcome physical barriers, South African law emphasises that physical accommodations should be provided to a person with a communication disability as highlighted in the *Criminal Procedure Act 51 of 1977 (CPA)*, which states that upon application by the state and in accordance with the provisions of the relevant sections in the CPA, such witnesses may testify in a room equipped with a closed-circuit television system.

South African law further provides for the appointment of an intermediary for a person with a communication disability, as highlighted in the *Criminal Law (Sexual Offences and Related Matters) Amendment Act 32 of 2007*. It is stated that:

[*W*]henever criminal proceedings are pending before any court and it appears to such court that it would expose any witness under the biological or mental age of eighteen years to undue mental stress or suffering if he or she testifies at such proceedings, the court may, subject to subsection (4), appoint a competent person as an intermediary in order to enable such witness to give his or her evidence through that intermediary.

Another procedural accommodation mentioned in the CPA relates to language accommodations, as it is recommended that the appointed intermediary for persons with communication disabilities should be conversant with the language of the witness. The use of sign language (and a qualified sign language interpreter), as well as other means of communication methods, should be provided for. In the CPA, Section 161(2) states that the expression ‘viva voce’ shall, in the case of a ‘deaf and dumb witness’ (terminology used in the Act), include sign language and, in the case of a witness younger than 18 years (including a mental age below 18 years), include demonstrations, using anatomical dolls, gestures or any other form of non-verbal expression.

Furthermore, *the Children’s Act 38 of 2005* (which applies to all children, including victims with communication disabilities who are younger than 18 years old and appearing in a children’s court) also mentions appropriate questioning techniques that may be used in the court system (this does not apply to the criminal courts). However, to date no specific guidelines have been developed as to how these differential questioning techniques should be employed (Carter & Boezaart, 2016).

For justice to be served for persons with disabilities, the South African criminal justice system must consider developing alternative methods that (1) enable witnesses with disabilities to fully partake as a witness, (2) include the admissibility of earlier statements made by the victims in place of their court testimony and (3) reduce the so-called discriminatory procedure of subjecting these witnesses to psychological examinations in an attempt to provide evidence that they are competent to give testimony (Pillay 2012). Evidentiary barriers were addressed in foreign law in Israel by the *Investigation and Testimony Procedural Act 2005*, which facilitates court testimony of persons with mental and cognitive disabilities – whether victim, witness or offender (Ziv 2007). The individual is allowed to give evidence in a modified court procedure and the Act requires that comprehensive accommodations be provided to persons with disabilities (Ziv 2007). However, Flynn (2016) cautions that the adaptation of the rules of evidence and procedures in criminal cases involving persons with disabilities may have the potential to be highly disputed.

Accommodating a witness with communication disabilities during the court process should be prioritised, as the evidence of such witness is usually essential for a successful conviction in the criminal court. It is particularly important that a fair trial process should be encouraged through the provision of additional supports, as well as through the adaptation of the rules of evidence and procedure (Benedet & Grant 2012). These accommodations are in line with the prescriptions of the CRPD, which specifically mentions in Article 13 that ‘procedural and age-appropriate accommodations’ should be provided to enable persons with communication disabilities to fully participate in the legal proceedings (Ortoleva 2011; United Nations 2006).

In summary, the aim of this research was to identify court accommodations, recommended by legal experts, that could assist individuals with severe communication disabilities to achieve justice in the South African court system.

## Research method and design

### Study design

A qualitative research design was used to conduct a discussion with a panel of legal experts (Creswell & Poth 2018; Diaby et al. 2015; Jensen et al. 2017). The expert panel was guided by a human rights framework that influenced the study framing, design, data collection and analysis (Skempes, Stucki & Bickenbach 2015).

### Participants in the study

Participants were selected using purposive, non-probability, expert sampling, which is a positive tool to use when investigating new research areas (Etikan, Musa & Alkassim 2016) – in this case, court accommodations for persons with communication disabilities. Ten potential participants were identified based on their professional experience of working with victims with communication and intellectual disabilities who had been victims of crime and the fact that they had worked with these individuals during the court process. Of the 10 potential participants, eight consented to partake in the expert panel discussion. Unfortunately, three experts were unable to physically attend because of unforeseen personal and logistical reasons, but as they recognised the value of the study, they inquired if they could do so remotely, in an asynchronous manner. To optimally benefit from their expertise, it was decided to collect their data via an email interview in which the exact questions that had been asked during the panel discussion were sent to them. Their responses were analysed and summarised and returned to them for verification as part of member checking. Thereafter, the first author presented their responses (with their consent) in the form of a PowerPoint presentation on the same day as the expert panel discussion. The other five experts attended and participated in the expert panel discussion that was hosted at a venue convenient for all involved. The participants’ biographical details are shown in Table 1. The participants all knew each other professionally, which led to rapport and trust being established quickly.

**TABLE 1:** Participant biographical details.

Expert number	Age	Gender	Language	Qualifications	Current title and role	Years’ experience	Specific expert experience
Expert 1	68 years	Female	English	B. Social Work B. (Hons) MA PhD	Consultant: Child Rights and Child Protection	30 years	Pre-court preparation therapy Post-court therapy for child victims (physical and sexual abuse) Assessment for the use of the intermediary system
Expert 2	61 years	Male	English	MA MSc PhD	Associate Professor and Principal Clinical Psychologist	25 years	Expert witness Reporting to court on various questions regarding rape complainants with intellectual disability
Expert 3	43 years	Female	Afrikaans	B. Iuris LLB Certificate in DNA evidence	State Advocate and Case Manager; Sexual Offences and Community Affairs Unit (NPA)	20 years	Public Prosecutor (District and Regional Court) State Advocate Case Manager Sexual Offences and Community Affairs Unit
Expert 4	61 years	Female	English	Nursing Sciences (Professional Nurse) B. Criminology (final year)	National Coordinator and Deputy Director: Government court preparation programme	25 years	Author of first Court Preparation Programme Researcher who piloted and institutionalised the Victim Impact Statements in the trial process
Expert 5	52 years	Female	Afrikaans English	B. Iuris LLB BA (Hons) LLM LLD	Associate Professor (previously Public Prosecutor)	28 years	Public Prosecutor for 5.5 years Prosecutor in specialised sexual offences court Published author of various manuscripts
Expert 6	61 years	Female	isiZulu English	M. (Clinical Psychology) PhD	Senior Lecturer Clinical Psychologist	18 years	Assessing survivors of sexual assault who have an intellectual disability
Expert 7	44 years	Female	English	M. Soc. Sci. (Clinical Psychology)	Principal Clinical Psychologist	18 years	Forensic mental health examinations of rape survivors with intellectual disabilities in terms of relevant legislation
Expert 8	63 years	Female	Guajarati English	M. (Mental Health) PhD	Director of NGO for abused children	30 years	Therapeutic intervention with child victims of abuse Forensic assessments for the courts

LLD: Doctor of Laws; BA: Bachelor of Arts; LLM: Masters of Laws; M.Soc. Sci: Master of Social Science; LLB: Bachelor of Laws; DNA: deoxyribonucleic acid; MA: Masters of Arts; Msc: Master of Science; PhD: Doctor of Philosophy; B. Social Work: Bachelor of Social Work; NGO: non-governmental degree; B. Hons: Honours degree.

Furthermore, all participants had experience of working with persons with disabilities during the legal process.

### Data collection

Before recruitment commenced, ethics approval was obtained from the Research Ethics Committee of the relevant institution. An email was sent to each participant with full details and instructions about the panel discussion. Once consent had been obtained from the participants, the programme for the full-day panel discussion was sent to them to allow adequate preparation and reflection time. At the beginning of the panel discussion, the researcher reiterated the topic, aim and purpose of the day. The procedure and timeline were highlighted. Experts were also reminded that their participation was voluntary and that they were allowed to discontinue at any given time without any negative consequences.

Prior to the expert panel discussion, the eight experts had been asked to prepare a presentation of 25–30 min on the invited topic to address the following questions: (1) Could you briefly discuss your experience with persons with communication disabilities in the criminal justice system? (2) Have you previously successfully asked for accommodations, and if so, can you please elaborate? The experts sent their presentations to the first author who acted as the primary correspondent and chair of the day. The first three presentations were presented by the first author. Each presentation provided a thought-provoking perspective on the invited topic (court accommodations for persons with communication disabilities), identified major trends and made suggestions for further accommodations. In the afternoon, a group discussion (similar to a focus group) followed, in which the following question was discussed: What may facilitate the process for a victim with a communication disability to be able to access and participate on an equal footing in the court system and process?

Apart from the audio recording, the third author also typed the full-day’s panel discussion to contribute to the trustworthiness of the data. She made a verbatim transcription of both the individual presentations and group discussion and then audited each transcript against the original audio recording. A total of 20% of the transcriptions were additionally checked by an independent researcher. Discrepancies were noted and revised when necessary (the formula used to calculate agreement: (Hallgren 2012). A 98% level of agreement was reached. This rigorous process greatly enhanced the procedural integrity of the transcripts (McLellan, MaCqueen & Neidig 2003).

### Data analysis

The researcher used ATLAS.ti 8, a computer-assisted qualitative data analysis software (CAQDAS), to conduct a thematic analysis and combined it with an inductive coding approach (Fereday & Muir-Cochrane 2006). Friese, Soratto and Pires (2018) describe seven phases of conducting a thematic analysis when using a CAQDAS to expand on Braun and Clarke’s (2006) six phases, namely, (1) becoming familiar with the data; (2) generating initial codes; (3) developing a structured code system; (4) searching for themes; (5) reviewing themes; (6) defining and naming themes; and (7) producing the report. This followed on first trying a deductive approach by using Article 13 (Access to Justice) of the CRPD as a coding framework. However, it proved to be an unreliable approach as a stable code structure could not be achieved (Friese et al. 2018).

The data were coded and analysed by the first author, after which authors 2 and 3 independently checked the codes and themes to increase inter-coder reliability and agreement of the data (Campbell et al. 2013). The process of initial coding (phase ii) resulted in a list of 244 codes. Next, a process of re-reading the coded segments, renaming, splitting and merging codes was conducted, which resulted in a total of 46 codes in the final structured code system (phase iii) (Friese et al. 2018).

### Ethical considerations

This article is part of one data source that is part of the first author’s PhD research where ethical clearance was obtained from the University of Pretoria, South Africa. Ethical Clearance number: GW20180718HS Student number: 29642630.

## Results

Table 2 shows the structured code system used in the study. The bold capital letters present category labels that serve as titles, and all data segments were distributed under the subcodes of a category (Friese et al. 2018). The number in the column ‘Grounded’ shows how frequently a code was applied.

**TABLE 2:** Structured code system.

Categories and subcodes	Definition of category and subcode	Grounded
**Accommodations:** Court accommodations, relevant services such as intermediaries or making the court accessible		**61**
• Alternative communication methods/strategies	Alternative ways of communicating in court by the witness, for example, AAC, the use of anatomical dolls and alternative strategies (e.g. simple questioning techniques)	17
• Intermediary services	Intermediaries and any services related to intermediaries	13
• Expert evidence	The need for and importance of expert evidence to be given in court for witnesses with disabilities	12
• Environment	Physical accommodations, for example, wheelchair access and environment adaptations such as a private testifying room (negative and positive examples were included)	10
• Expert support person	A lay or legal assessor to support the magistrate during legal proceedings	5
• Victim impact statements	Explanation and importance of victim impact statements and how they can be used	4
**Court preparation programmes:** Court preparation offered by government or non-governmental organisations (NGOs)		**33**
• NGO 1	Process of the court preparation programme at NGO 1	12
• Government	Process of the NPA’s Ke Bona Lesedi Court Preparation Programme	11
• NGO 2	Process and description of the court preparation programme at NGO 2	7
• Purpose	Purpose of court preparation for the victim and all involved	3
**Court system**: Court system and processes, for witnesses and professionals		**44**
• Equality	Highlighting the term ‘equality’ in the court system. Persons with disabilities should have the same (equal) rights as their peers and be able to access the court on an equal footing	13
• Challenges	Challenges to access the court system, and the challenges related to the rigid and inflexible procedures and processes that the courts follow	12
• Unrealistic expectations of victims	The court and court officials have unrealistic expectations of the victims with disabilities	9
• Process	The court processes followed (current as well as past processes)	6
• Trust of victims and families in process	Lack of faith in the court system by families and victims who did not find the system beneficial to pursue	4
**LAW:** Specific law regarding access to the court system for persons with disabilities		32
• Specific legislation for persons with disabilities	Specific mention of laws and policies for persons with disabilities, nationally and foreign law	**15**
• Reform	Mention of law reform and the importance of law reform	12
• Challenges	Challenges of the law, for example, law is perceived as dichotomous, which could disempower persons with disabilities	5
**Professional experience:** Professionals involved in the court process, either on a professional or personal level. Also, statements on how the professional interacts with or responds to the victim with a disability (lack of patience)		**95**
• Specific training needed	Specific training of professionals who work with persons with disabilities to address aspects such as knowledge, awareness and patience	36
• Responsibilities	Responsibilities of specific professionals in the court system, for example, the prosecutor, social worker and police.	23
• Importance of training	Importance of training of professionals so that victims could access the court system in a fair manner	21
• Work challenges	Challenges faced by professionals in the court system – being overworked, having too large caseloads, etc. This results in witnesses with a disability not being able to fully access the court system	15
**Witness:** Comments linked directly to the victim or witness with a communication disability		**149**
• Level of disability	The type or level of disability of the witness (e.g. intellectual disability and physical disability) and how the level of disability affected the victim’s ability to consent to sexual intercourse	29
• Personal factors	Personal factors related to the witness (language barriers, self-blame, protecting the perpetrator, etc.) that have an impact on his or her access to the court system	23
• Witness competency	Basic competency, truth–lie competency and the ability of the person with a communication disability to testify in court and be a witness	23
• Human rights	Examples of human rights violations affecting the victim’s human dignity and equality; no human respect for the witness or victim	20
• Environmental factors	Any processes or persons other than the witness’ family mentioned in a negative way that prevented the witness from accessing the court system effectively	19
• Tools, assessments and methods used	Tools, models, assessments and the processes currently used with witnesses in South Africa	16
• Support services	Importance of support services for the witness	7
• Unfair discrimination	Unfair discrimination experienced by the witness	7
• Family	The witness’ family	5

AAC, alternative and augmentative communication; NPA, National Prosecuting Authority.

Table 3 provides examples of codes (specific quotes from the experts) that emerged from the six main categories.

**TABLE 3:** Examples of codes (quotes) in specific categories.

Categories	Codes based on verbatim quotes from participants
Accommodations	‘We need non-verbal ways of communication that are reliable and valid’.
Court preparation programmes	‘It is furthermore the process of empowering the witness or the complainant by familiarising them with information regarding the court environments so that they are not afraid of the unknown, what are they going to face, who they going to face, where they going to face and court processes, legal process and legal terminology and it all has to be age appropriate and how do we address that in terms of their disability? and it’s very helpful when we get a report on what type of disability? what their medical, their mental functioning is, so that you can address that witness or the complainant on that level’.
Court system	‘We need a more flexible court system that shows its understanding of the witness’ disability and tries to work with [*her*] to enable optimal testimony’. ‘We need a court system that is disability-friendly, and I don’t believe that our present system is so, especially as it relates to intellectual disability’.
Law	‘The Criminal Procedure Act 51 of 1977 (the CPA) provides for a number of protective measures for child and adult witnesses as well as witnesses with disabilities’. ‘The PEPUDA act, in section nine and in section six says ‘no person may unfairly discriminate against any person on the grounds of disability including denying or removing from any person who has a disability, any supporting or enabling facility necessary for their functioning in society and in court’.
Professional experience	‘Training is critical’. ‘There is a need for ongoing training’. ‘…..training, training, training. And I think that we need to see training as never ending, we can’t do training in March and then leave it for another two years. We just have to keep training’.
Witness	‘With mental disability I have encouraged police/prosecutors and sometimes testified in court, to understand the nature of the disability and how it impacts on the child and evidence. Sometimes I have not been successful and sometimes when the mental disability is profound, the child is unable to describe the offence and then the case only proceeds where there is other evidence e.g. – DNA or a witness’.

*Promotion of Equality and Prevention of Unfair Discrimination Act*, 2000 (PEPUDA); deoxyribonucleic acid (DNA).

**FIGURE 1:**
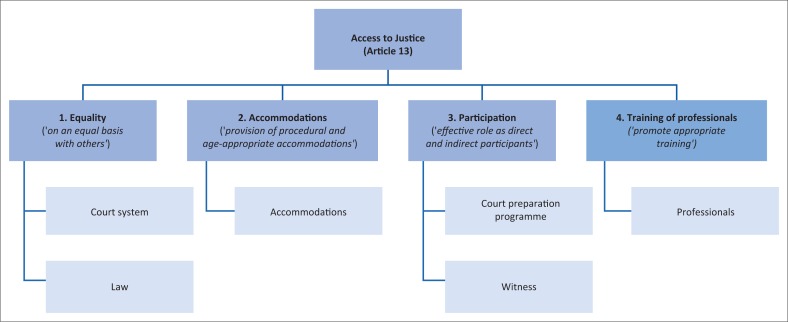
Conceptual framework, themes and categories.

Next, the authors used Article 13 (Access to Justice) of the CRPD (United Nations 2006) as a conceptual framework to link categories to themes (Drew et al. 2011; Harpur 2012). Four main themes were identified, namely, equality, accommodations, participation and training of professionals. The themes and related categories are presented in Figure 1.

## Discussion

An in-depth discussion of the four themes – equality, accommodations, participation and training of professionals – is presented here.

### Equality

Article 13 specifically mentions the importance of ensuring access to justice for persons with communication disabilities on an ‘equal basis’ with others (United Nations 2006). The South African court system is not always considered beneficial or easy to pursue as one expert highlighted:

…[*P*]eople not seeing any value in the criminal justice system because the legal system has never actually benefited them in any way, the whole process of trying to go through the system is just one more big obstacle… impenetrable obstacle! The Criminal Law (Sexual Offences and Related Matters) Amendment Act 32 of 2007, Section 170A, Subsection (1). (p. 106)

If transformative equality is to be achieved, processes and procedures within the court system need to be adapted and modified to enable persons with communication disabilities to participate equally in court. The court and criminal justice system have an important role to play in furthering transformative equality. In order to ensure that it promotes its aims of protecting vulnerable groups such as persons with communication disabilities, the court system is compelled to develop certain criteria to accommodate witnesses with communication disabilities (Fredman 2005; Lord & Brown 2011).

The CRPD recognises that laws are not always sufficient to protect the rights of persons with disabilities, and therefore, strategic litigation and law reform are needed to ensure that laws are in line with international human rights standards such as the CRPD (Drew et al. 2011; Flynn 2013). Some countries have laws that protect and assist witnesses with disabilities to access the court system on an equal basis and have set a benchmark for other countries, for instance, Scotland’s *Vulnerable Witnesses Act* of 2004, Israel’s *Investigation and Testimony Procedural Act (Accommodations for People with Cognitive or Mental Disability) of 2005* and India’s *Rights of Persons with Disabilities* Act of *2016*. Yet, the development of policies and laws historically excluded persons with communication disabilities, which implies that their needs were not adequately addressed. According to Drew et al. (2011), it is therefore essential that persons with communication disabilities are actively involved in the law reform process.

### Accommodations

The court has a responsibility to ensure fair and equal access for all witnesses, including those with communication disabilities, and certain procedural accommodations could assist the court in achieving transformative equality. When discussing types of accommodations, Msipa (2015) puts forward the following strong statement:

In the criminal trial setting, the question should not be whether a person is competent to testify; rather it should be what types of accommodations are required to enable the person to give effective testimony? (p. 89)

The CRPD specifically mentions that provision of procedural and age-appropriate accommodations should be provided to a witness with a communication disability in order to ensure his or her effective access to justice (United Nations 2006).

#### Lay or Legal Assessors

Section 34 of the *Magistrates’ Courts Act 32 of 1944* allows for the appointment of assessors in both criminal and civil cases in South Africa. Expert assessors are generally experienced people in law who are advocates or magistrates (Department of Justice and Constitutional Development 2019). Lerm (2012) explains the rationale for this practice, namely, to assist magistrates and judges who are only professionally trained and who frequently lack the expertise and practical knowledge to match that of the experts who would testify in cases before them. Therefore, the use of expert assessors to assist judges and strengthen their competence to judge complex matters was developed. Appointing a legal assessor who is a trained and skilled expert in communication disability could assist the judge or magistrate to understand the witness’ disability, as well as the accommodations that are needed to support this witness to be able to participate and testify in court.

#### Intermediaries

As criminal proceedings in court are generally not disability-friendly, intermediaries are used to assist both witnesses and perpetrators with communication disabilities during the court process and ultimately to support the witness or perpetrator to participate equally in the court process. This process is similar to the appointment of intermediaries in criminal cases for all witnesses under the biological or mental age of 18 years. An intermediary is a facilitator who assists a witness to testify and give evidence in court. As a result, all communication interaction exchanged between the witness and the court takes place through the intermediary, including examination-in-chief, cross-examination and re-examination (Fambasayi & Koraan 2018). The role of the intermediary is to translate the questions from the prosecution and the defence attorney and put them to the witness in a language and terminology that the witness understands (Jonker & Swanzen 2007).

Foreign case law in England has allowed the intermediary to assist with questions for cross-examination of the witness, which had been agreed in advance by all parties involved (R v Michael Boxer [2015] EWCA Crim 1684) (The Advocate’s Gateway 2019). This is a strategy that could assist the courts with regard to the cross-examination from the defence.

#### Alternative and Augmentative Communication

Alternative and augmentative communication (AAC) strategies and techniques are used by individuals with significant communication disabilities who cannot rely on spoken language alone for communication purposes, for example, persons with cerebral palsy or those with intellectual disability (Beukelman & Mirenda 2013). Broadly, AAC systems have a binary taxonomy that distinguishes between unaided and aided communication systems. In the case of unaided communication, persons use only their bodies to convey their messages, for example, systems with linguistic features such as a formal sign language (e.g. South African Sign Language [SASL] and finger spelling) or systems without linguistic features such as natural gestures, facial expressions and vocalisations (Beukelman & Mirenda 2013). In South African courts, persons with communication disabilities have been allowed to use unaided communication systems such as informal signs to testify in court (R v Ranikolo 1954 (3) SA 255 (0)). However, for many persons with severe communication disabilities, for example, those with significant physical disabilities and limited movement, the use of unaided communication systems (such as SASL) is not possible.

Aided communication can be defined as systems that require external assistance (e.g. using pictures or objects) to produce a message. As with unaided systems, aided systems also fall on the continuum of linguistic features. On the one end of the continuum, there would be symbol *sets* (*without* linguistic features), and on the other end, there would be symbol *systems* (*with* linguistic features) (Bornman & Tönsing 2019). Traditional orthography (e.g. letters of the alphabet) is an example of an aided symbol system with linguistic features that would allow literate individuals with a communication disability to generate their own messages. Alphabet letters can also be presented in Braille or Morse code format. Braille, a tactile symbol system for reading and writing that is typically used by blind persons, also requires literacy skills and hence the theoretical argument reverts to the issue of the literacy level of individuals with disabilities (Groce & Bakshi 2009; Statistics South Africa 2012). Unfortunately, using aided systems with linguistic features to testify is not applicable to the majority of South Africans with communication disabilities because of the notoriously high illiteracy rates in the local population (Groce & Bakshi 2009; Statistics South Africa 2012).

Blissymbols are a conceptually based graphic symbol system with linguistic rules and markers (Beukelman & Mirenda 2013). Blissymbols are placed half-way on the aided communication continuum between symbol sets with no linguistic features and symbol systems with linguistic features. Bliss Symbols have been used successfully in a South African court case (Toefy 1994). Unfortunately, Bliss Symbols are not commonly used in South Africa as part of the education system.

The other end of the aided communication continuum consists of symbol sets that contain finite numbers of easily guessable symbols with limited linguistic features. Symbol sets thus consist of a defined number of symbols that have no rules for expansion or generating new words, for example, Picture Communication Symbols (PCS). This means that messages can only be compiled by selecting symbols from the pre-selected set (Beukelman & Mirenda 2013). Symbol sets are particularly useful for non-literate persons, persons with limited literacy skills and preliterate persons. Preliterate persons (young children who have not yet acquired literacy skills or individuals who have not yet been exposed to literacy and who might still acquire literacy skills) often use graphic symbol sets that do not have linguistic features and therefore do not require literacy skills. It is important for preliterate individuals with communication disabilities to have access to alternative means to represent messages and concepts to communicate (Drager, Light & McNaughton 2010).

Therefore, aided AAC systems that do not have linguistic features, such as PCS, may be a viable option in the criminal justice system. For non-literate and preliterate individuals, the vocabulary required to access the court system could be selected and represented in the form of line drawings that could be displayed as a communication board or book. Alternatively, the required vocabulary could be programmed into a specific speech-generating device such as a tablet with specific AAC software (Caron, Light & Drager 2016; White et al. 2015).

These systems could assist non-literate, minimally literate as well as preliterate persons with communication disabilities to participate with others in their environment, as the meanings of many of the symbols and line drawings are easy to understand (Dada, Huguet & Bornman 2013). The use of systems with a set of pre-selected vocabulary in the court system also has specific implications. The vocabulary will be selected from a pre-determined symbol set, and thus it will not be generated, as would have been possible when a symbol system such as traditional orthography or Braille had been used. These implications could be remedied by adding multiple foils and categories in the pre-determined symbol set (White et al. 2015).

In countries such as England, Wales, Northern Ireland and Scotland, witnesses with communication difficulties are permitted to use both aided and unaided forms of AAC to support their testimony (O’Leary & Feely 2018). The South African court system needs to formally recognise AAC as a form of communication and giving testimony for witnesses with communication disabilities, and provided that the court procedures and rules of evidence are not undermined, this form of accommodation should be allowed in court (Flynn 2016).

### Participation

The CRPD, and specifically Article 13, highlights the importance of persons with disabilities being active participants as witnesses in the court process (United Nations 2006). In South Africa, the government and non-profit organisations offer multiple court preparation programmes to empower the witness with disabilities to participate effectively in the court system. Greater awareness needs to be raised and wider education be offered regarding the relevant court preparation programmes so that persons with disabilities and their families would know whom they can turn to when wanting to access the court system.

The purpose of the Ke Bona Lesedi Court Preparation component offered by the National Prosecuting Authority of South Africa (NPA) is to prepare and empower victims with communication disability (witnesses and their families) for testimony (Tewson 2017). This skilled and practical intervention is prosecutor guided and aims to empower witnesses to give credible evidence in court. The court preparation officers (CPOs) accompany the witnesses and complainants from beginning to end, encouraging them, teaching them coping mechanisms, referring them for counselling and giving crucial feedback to the prosecutor. They also ensure that the prosecutor knows how to approach a witness with specific communication needs (Tewson 2017). Court preparation officers, together with the intermediaries, play a critical role in the court process and their role should be advocated in all courts as part of ensuring equal access to justice for witnesses with communication disabilities. Court preparation officers identify the accommodations and special needs of the witness prior to testimony and consultation with the prosecutor, which ensures that the necessary accommodations are timeously arranged (Tewson 2017).

A barrier and recurring obstruction to witness participation is the victim’s level of disability and ability to be a competent witness. Pillay (2012) strongly argues that every attempt must be made to find reasons why witnesses with intellectual disabilities should be permitted to give evidence, rather than why they should *not* be allowed to testify. Scottish Law has addressed this barrier where the *Vulnerable Witnesses (Scotland) Act of 2004* legally removed the competence test for vulnerable witnesses. The advantage of removing this test is that it allows the magistrate to determine the witness’ reliability, rather than to rely on a test that does not necessarily ensure the truthfulness of their evidence. It also ensures that victims with communication disabilities have the opportunity to be heard (Turner, Forrest & Bennett 2016).

### Training of professionals

The CRPD specifically mentions the importance of training all professionals who work in the court system.

Lack of training is consistently labelled as a barrier in the South African court system as it gives rise to, for example, lack of awareness, lack of patience and lack of knowledge (Bornman et al. 2016).

This type of training has been demonstrated to be effective. For example, a Swedish study that focused on the training of active crime investigators of alleged child abuse who participated in six different half-year courses between 2007 and 2010 showed effective outcomes in shaping the interviewers’ behaviour towards better compliance with foreign recognised guidelines (Cederborg et al. 2013). This is just one of many examples of the benefits of specific training programmes for legal professionals. Access to justice can be improved when these professionals can receive the relevant training (Larson 2014), and this practice should be prioritised in the South African court system.

## Evaluation of study

This study focused on the South African court system and therefore included only South African legal experts.

An expert panel incorporating foreign experts could have added a more global perspective on the accommodations needed for persons with disabilities. A comparison between South African and foreign experts should be considered for future research to obtain a more comprehensive list of possible accommodations that have demonstrated effect. Other professional stakeholders (therapists, parents and caregivers) could have also been included in the expert panel to provide additional accommodations.

Although this study focused predominantly on the witness and victim, the same supports could be offered to perpetrators and offenders too. Effective access to justice could also be achieved, the integrity of the court system could be maintained (Flynn 2016) and all human rights would be uplifted.

This study focused on the CRPD as a human rights framework, although future research could also include other relevant frameworks such as the International Classification of Functioning, Disability and Health (ICF) when aiming to identify possible court accommodations for persons with communication disabilities.

Furthermore, when conducting legal research and making legal statements, a systematic literature search approach (such as a legal systematic review) could be followed (Baude, Chilton & Malani 2017). Therefore, future research could focus on conducting systematic legal reviews that are evidence-based to determine a scope of published literature that focuses on globally accepted court accommodations for persons with communication disabilities.

## Conclusion

The aim of this study was to identify court accommodations that could assist persons with communication disabilities to participate in the court system. The reality is that persons with communication disabilities who were victims of crime, as well as their families, still face many barriers when accessing the court system. As a result, they sometimes choose not to report the victimisation, as all too often this process seems to be more of an obstacle than a benefit. Similarly, perpetrators with communication disabilities may experience profound disadvantages in preparing and presenting their defence if not provided with appropriate accommodations during both the pre-trial and trial processes.

Foreign and national laws forbid discrimination against persons with communication disabilities and insist that they should be given fair and equal access to the court system. For transformative equality to be achieved, certain rules and laws need to be changed to include specific accommodations for witnesses with communication disabilities so as to enable them to participate effectively in the court system. Furthermore, it is also the responsibility of the courts to ensure effective access to justice. Participation in court processes can benefit both the victim and the perpetrator in many ways because it will allow them to tell their version of events and feel believed. More importantly, it may assist these individuals to experience the effective fulfilment of their human rights.
